# From Porous to Dense Nanostructured β-Ti alloys through High-Pressure Torsion

**DOI:** 10.1038/s41598-017-13074-z

**Published:** 2017-10-19

**Authors:** Conrado R. M. Afonso, Angelica Amigó, Vladimir Stolyarov, Dmitri Gunderov, Vicente Amigó

**Affiliations:** 1 Department of Materials Engineering (DEMa), Universidade Federal de São Carlos (UFSCar), Rod. Washington Luis, km 235, 13565-905 Sao Carlos-SP, Brazil; 2 Institut of Materials Technology (ITM), Universitat Politècnica de València (UPV), Camí de Vera s/n, 46022 Valencia, Spain; 3 Mechanical Engineering Research Institute of RAS, Moscow, 101990 Russia; 4 National Research Nuclear University MEPhI, Moscow Engineering Physics Institute, Moscow, 115409 Russia; 5 Saint Petersburg State University, Saint Petersburg, 198504 Russia

## Abstract

β-Ti alloys have low elastic modulus, good specific strength and high corrosion resistance for biomaterial applications. Noble elements, such as Nb, Ta and Mo, are used to obtain β-Ti due to their chemical biocompatibility. However, due to their refractory nature, β-Ti requires specific processing routes. Powder metallurgy (P/M) allows for the development of new β-Ti alloys with decreasing costs, but dealing with high-elemental-content alloys can lead to a lack of diffusion and grain growth. One method to refine the structure and improve mechanical properties is a severe plastic deformation technique through high-pressure torsion (HPT). The aim of this work was to evaluate the conversion of P/M porous β-Ti-35Nb-10Ta-*x*Fe alloys to dense nanostructures through high-pressure torsion in one deformation step and the influence of the structure variation on the properties and microstructure. TEM analysis and ASTAR crystallographic mapping was utilized to characterize the nanostructures, and the properties of P/M β Ti-35Nb-10Ta-xFe alloys processed by HPT were compared. The initial microstructure consisted mainly by the β-Ti phase with some α-Ti phase at the grain boundaries. The HPT process refined the microstructure from 50 µm (P/M) down to nanostructured grains of approximately 50 nm.

## Introduction

To obtain advanced biomaterials with low elastic modulus and acceptable mechanical strength, titanium alloys can be made with a high percentage of refractory materials, such as niobium, tantalum and molybdenum. However, high amounts of these materials can make the manufacturing processes of these alloys difficult^1–3^. One route to obtain β-Ti alloys is powder metallurgy (P/M), which allows one to obtain customized alloys. However, this method presents intrinsic limitations, such as porosity, a lack of diffusion and an increased grain size with various sintering parameters^4,5^. Ti-based alloys have been widely used as biomaterials for high-load implants due to their high specific strength, good biocompatibility, variety of processing routes and superior corrosion resistance^6–8^. Manufacturing β-Ti alloys for biomaterial applications usually involves the addition of isomorphous Nb, Ta, Mo and Zr, a eutectoid (Fe and Sn) β-stabilizer and biocompatible elements^8–11^.

The conventional route to produce and process these β-Ti alloys involves casting (arc melting, vacuum induction melting or skull casting) and subsequent thermomechanical treatments^3,10,12^. Alternatively, β-Ti alloys composed of noble elements and high-melting-point alloying elements can be produced by P/M, with advantages in near net shaping, microstructure homogeneity and porosity control by varying the sintering parameters^4,5^. To significantly refine the microstructure of β-Ti alloys, usually to a grain size in the range of tens to hundreds of microns, a severe plastic deformation (SPD) route, such as high-pressure torsion (HPT), which applies a high-pressure and concurrent torsion strain, can be very efficient. HPT may be a potential processing method to eliminate porosity resulting from previous P/M routes. Then, the elastic modulus and mechanical properties can be optimized by introducing a high density of dislocations^13–15^.

For the characterization of nanocrystalline phases (such as the ones observed in β-Ti alloys and HPT-processed samples of metallic materials) and their crystallography, automatic crystal orientation measurements (ACOM-TEM) with an ASTAR system might be a powerful technique^16,17^. An ASTAR system (to unscramble) crystalline structure of the nanophases, can provide grain size, crystallographic orientation (texture) and, finally, hundreds of thousands of electron diffraction spot patterns (NBD: nanobeam diffraction) generated in minutes or a few hours. The objective of this work is to evaluate the conversion of P/M porous β-Ti-35Nb-10Ta-*x*Fe alloys to dense nanostructures through high-pressure torsion in one deformation step and the influence of the structure variation on the properties and microstructure analyzed by advanced TEM characterization.

## Experimental Procedures

Powder metallurgy of elemental blended powders was used to obtain the alloys. The nominal compositions of β-Ti were: Ti35Nb10Ta and Ti35Nb10Ta3Fe (wt%). Pure metal powders were supplied by Atlantic Equipment Engineers in high purity and different sizes: Ti (99.7% purity, 55 µm average size), Nb (99.8%, 20 µm), Ta (99.8%, 8 µm) and Fe (99.9%, 34 µm). Compaction was applied in a floating matrix with a diameter of 20 mm to obtain a 5 mm thick sample, followed by a sintering procedure described elsewhere^16^. Microstructure characterization was performed with a field emission gun (FEG) scanning electron microscope (SEM, Zeiss Ultra 55) using backscattered electrons (BSE) to reveal the microstructure and obtain grain size measurements by image analysis. X-ray diffraction (XRD) (PANalytical cubicPro) was used to determine the phases. For HPT processing, the disc sample was positioned between two anvils, which applied a compressive pressure of 6 GPa and simultaneously produced a torsional strain, for 5 complete revolutions. The HPT setup had an anvil with a groove allowing for the production of disc-shaped samples of 20 × 0.7 mm in size. TEM characterization was performed on ½ R, where R is the radius of the HPT disc. Oxygen measurements were made using a LECO 400 series instrument. Nanohardness tests were conducted with an MTS Nano 200 G using CSM mode with deep control, indenting 1600 nm (1.6 μm) of depth, to obtain the hardness and elastic modulus. Automatic crystal orientation mapping (ACOM) was acquired using a JEOL JEM 2100 F (TEM/STEM) with an acceleration voltage of 200 kV with a field emission gun (FEG) using an ASTAR^TM^ NanoMegas system^17,18^. The mapping step size was 5 nm based on a rectangular grid (600 × 400 pixels).

## Results and Discussion

**Figure 1:**
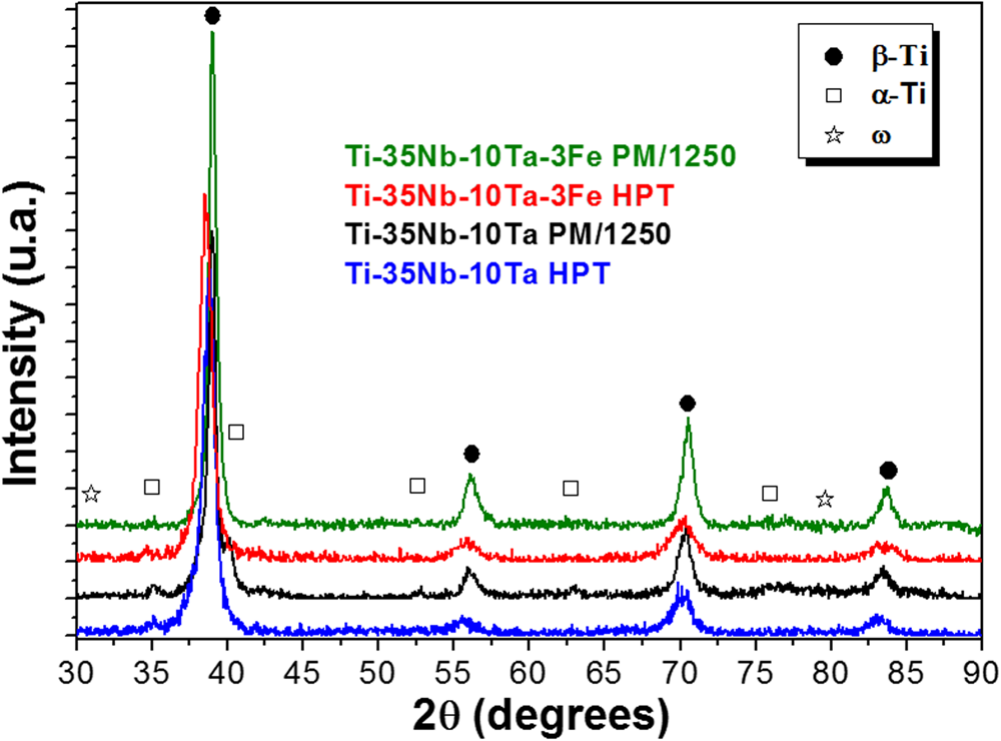
XRD patterns of Ti35Nb10Ta and Ti35Nb10Ta3Fe sintered at 1250 °C and then processed by HPT at room temperature (5 revolutions) and P = 6 GPa.

The X-ray diffraction (XRD) patterns of the P/M Ti35Nb10Ta and Ti35Nb10Ta3Fe alloys sintered at 1250 °C and then processed by HPT at room temperature (5 revolutions) and P = 6 GPa mainly shows the presence of the β-Ti phase, with low intensity peaks of the α-Ti phase (Fig. 1). The β-Ti (bcc) and α-Ti (hcp) peaks of the P/m samples become broader after HPT, indicating a considerable reduction of grain size due to severe plastic deformation. The intensities of the α phase peaks (and volume fraction) decrease with the addition of Fe, showing higher stabilization of the β phase, under the same conditions for the sintering process. Wei *et al*.^19^ confirmed that, for the addition of oxygen from 0.3% up to 1.8%, there is no considerable change from the stabilized β-Ti phase for TNZT alloys. Therefore, in this work, the measured oxygen content of 0.7% itself might not cause considerable changes to the Ti35Nb10Ta(3Fe) alloys.

**Figure 2:**
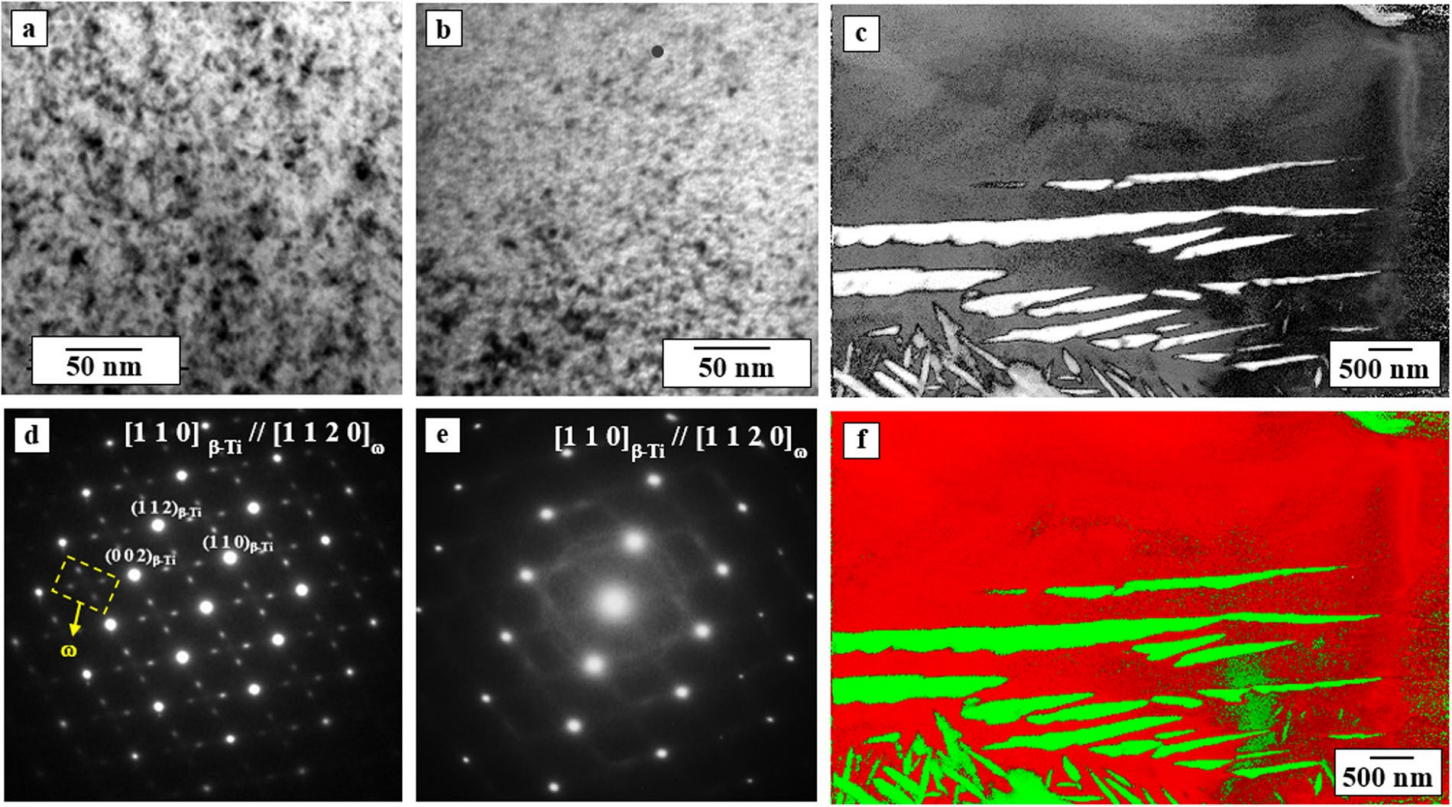
TEM micrographs in (**a**) bright field (BF) mode showing the ω-Ti nanoprecipitates dispersed in β-Ti grain in the P/M samples of the Ti35Nb10Ta alloy and (**c**) for the Ti35Nb10Ta3Fe alloy, both sintered at 1250 °C, together with the respective (**b**) and (**d**) SAD patterns of the β-Ti region with an orientation relationship [1 1 0]_β-Ti_//[1 1 2 0]_ω_ between the β-Ti matrix and metastable ω phase for both alloys. ACOM map of The i35Nb10Ta alloy sintered at 1250 °C showing (**e**) virtual bright field (VBF) of the α + β region and (**f**) PhaseMap combined with a Virtual-BF image of the α precipitates (green) dispersed through the β-Ti (red) matrix.

The microstructure of the P/M Ti35Nb10Ta alloy sintered at 1250 °C is composed of β-Ti grains with sizes of approximately 45 μm and submicron α-Ti laths. The TEM analysis confirms the presence of a nanometric metastable ω phase in the P/M sample before HPT processing of the Ti35Nb10Ta alloy sintered at 1250 °C (Fig. 2a). The selected area diffraction (SAD) pattern performed on the β-Ti-rich region is shown in Fig. 2d, with [1 1 0]_β-Ti_/[1 1 2 0]_ω_ zone axes and double diffraction spots of the metastable ω phase. The P/M alloy with Fe addition (Ti35Nb10Ta3Fe) shows greater stabilization of the β-Ti phase, which is confirmed by the lower intensities of the α-Ti phase peaks in the XRD pattern (green). Additionally, the TEM image in Fig. 2c shows that the metastable ω phase precipitation remains but with a smaller fraction and finer nanometric scale. The corresponding SAD pattern for the P/M Ti35Nb10Ta3Fe alloy in the β-Ti-rich region is shown in Fig. 2e, confirming diffuse spots related to ω phase with the same orientation relationship [1 1 0]_β-Ti_//[1 1 2 0]_ω_ as that for the Ti35Nb10Ta alloy.

Figure 2c shows the automatic crystallographic orientation mapping (ACOM) image using ASTAR of the P/M Ti35Nb10Ta alloy sintered at 1250 °C with a virtual bright field (VBF) of the α + β region, and Fig. 2f shows the PhaseMap combined with the VBF of the β-Ti (red) and α (green) phases. The β-Ti phase is mainly observed, with some α + β regions (β-stabilizer-poor elemental concentration) and β-Ti-rich regions with Fe addition and a higher sintering temperature. Although the nanometric ω phase was observed within the β-Ti phase using TEM analysis (Fig. 2), the ASTAR provides complementary information on both the phase constitution and orientation distribution of the nanosized α laths dispersed in the β-stabilizer-poor regions (α + β region).

Although the XRD patterns show only α-Ti and β-Ti phases without clear peaks attributable to the metastable ω phase, the TEM analysis coupled with EDS analysis shows variation of the Nb content in the range of 27 to 35 wt% Nb (and other alloying elements, Ta and Fe), which can allow for metastable ω phase precipitation at a lower Nb content. Some works in the literature have indicated that the formation of the metastable ω phase can be favored by lower the cooling rates from the β-phase field, depending on the composition^20^.

**Figure 3:**
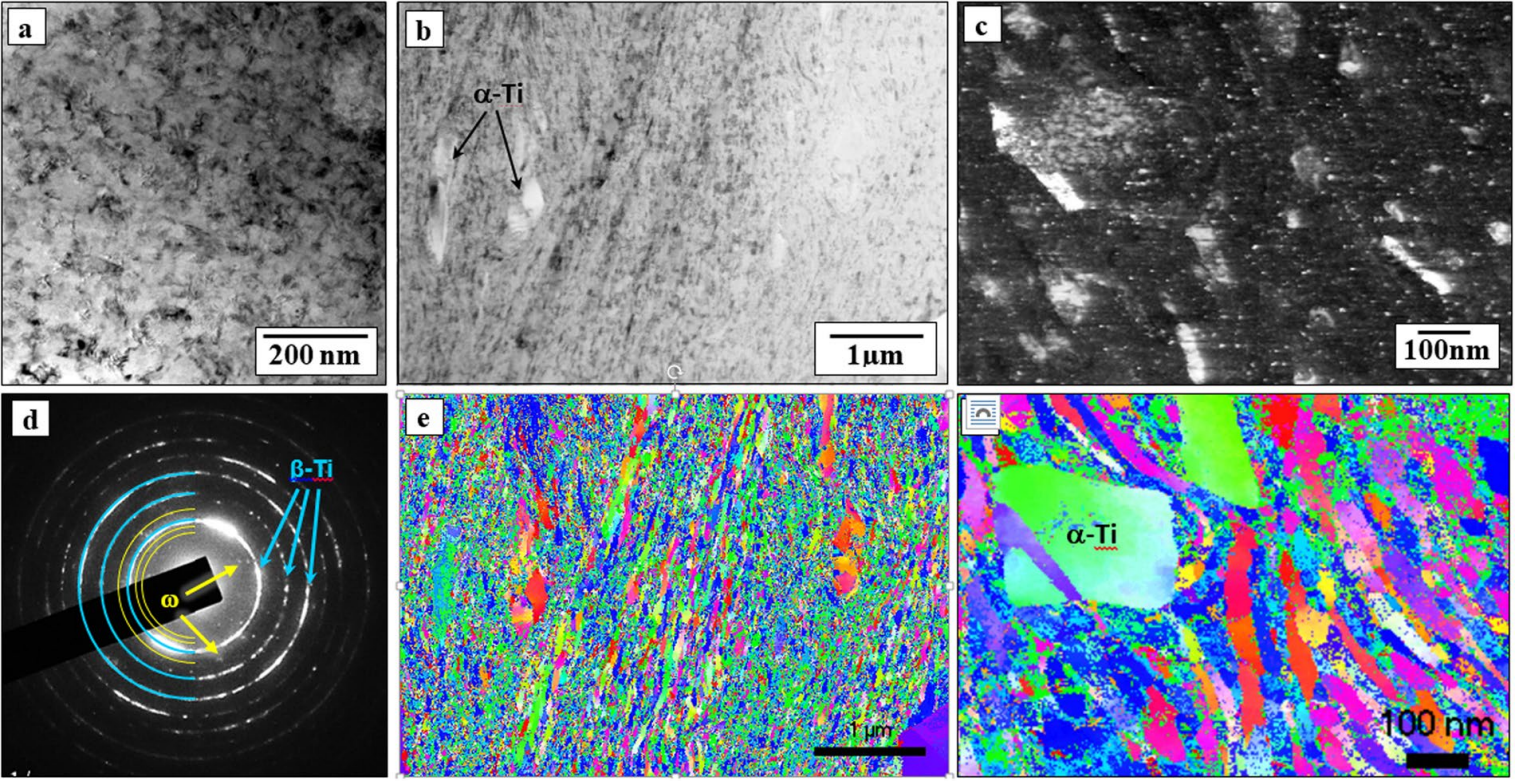
TEM micrograph in (**a**) bright field (BF) mode of the nanocrystalline β Ti35Nb10Ta alloy after HPT processing and the respective (**b**) SAD ring pattern typical of nanostructured materials with aleatory orientation. ACOM analysis of the HPT sample of the Ti35Nb10Ta alloy with (**c**) virtual bright field (VBF) of the nanocrystalline α + β region and (**d**) IPF-z orientation image showing a general view of the nanostructure. Higher magnification ACOM analysis showing in detail (**e**) the virtual dark field (VDF) of the ω precipitates dispersed through the nanocrystalline β-Ti grains and (**f**) IPF-z orientation image showing smaller soft β nanograins and α precipitates as well.

The TEM micrograph reveals (Fig. 3a and b), after HPT processing of the P/M sample, the resulting nanometric grain size of the β-Ti35Nb10Ta alloy obtained by severe plastic deformation. The TEM micrograph in Fig. 3a shows that HPT processing leads to a considerable reduction of the grain size due to the high deformation level imposed by the severe plastic deformation, which results in nanoscale grain size of the β-Ti phase^3,13–15^. The occurrence of nanocrystalline grains of mostly the β-Ti phase (light blue lines) is confirmed by the ring-shaped selected area diffraction (SAD) pattern, as shown in Fig. 3d, with additional spots related to the α and ω phases (yellow lines). Moreover, HPT of the α-Ti phase (harder) resulted in less fragmentation and reduction of the particle size than that of the β-Ti soft matrix due to the differences of mechanical strength between these phases. Although this is confirmed from the nanocrystalline SAD ring pattern, the nanograin boundaries and grain size cannot be clearly observed from conventional TEM imaging (Fig. 3a).

Figure 3b presents the ACOM analysis with the VBF and IPF-z crystallographic orientation micrographs of the nanocrystalline β-Ti grains for the Ti35Nb10Ta alloy after HPT processing. HPT leads to a grain size refinement from the 45 µm (P/M) grains of the Ti35Nb10Ta alloy sintered at 1250 °C to nanosized grains of approximately 50 nm (Table 1), decreasing the grain size by 3 orders of magnitude, which is very significant considering only the single deformation step. Figure 3c illustrates the higher magnification ACOM with the virtual dark field (VDF) image and the occurrence of very fine ω precipitates (white dots) dispersed throughout the nanocrystalline grains of the β-Ti matrix and some dispersion of hard α-Ti nanoprecipitates. Figure 3f shows the respective IPF-z crystallographic orientation micrographs at higher magnification.

**Table 1:** Variation of the porosity (%P) and grain size (µm) with the composition of the Ti35Nb10TaxFe alloys for P/M samples sintered at 1250 ^o^C and discs obtained after HPT deformation step (5 turns and pressure of 6 GPa).

Composition	Porosity (%P)		Grain size (µm)	
	PM/1250 °C	HPT	PM/1250 °C	HPT
Ti35Nb10Ta	5.1 ± 0.6	~0	45 ± 2	0.05 ± 0.02
Ti35Nb10Ta3Fe	9.2 ± 0.3	~0	60 ± 3	0.07 ± 0.02

The grain size measurements show an increase with the addition of Fe for the P/M samples, from 45 to 60 µm, according to the results in Table 1. The porosity increase from %P = 5.1% to 9.2% with the addition of 3 wt.% Fe confirms that Fe diffusion into the Ti crystalline structure is much higher than the opposite, and even with higher stabilization of the β-Ti alloy with Fe addition, it has a negative impact on the porosity and microstructure^22,23^. Such a combination of compositional variation along microstructure and the β-Ti phase composition lead to elastic modulus values of 82 to 98 GPa for the P/M samples of the Ti35Nb10Ta and Ti35Nb10Ta3Fe alloys (Table 2), respectively. These values are higher than those of the cast Ti35Nb7.5Ta (E = 65 GPa) and Ti35Nb3Fe alloys (E = 62 GPa)^20,21^. Table 2 shows that the elastic modulus decreases after HPT for the Ti35Nb10Ta (84 to 79 GPa) and Ti35Nb10Ta3Fe alloys (98 to 84 GPa). The HPT process can lead to an occurrence of deformation twins that act as a strengthening mechanism to improve the critical stress required for dislocation slippage. On the other hand, the pre-existing metastable ω precipitates induced by severe deformation by HPT can induce elastic softening^15,17^.

**Table 2:** Nanohardness (GPa) and elastic modulus (GPa) values for the P/M samples analyzed at different radial distances from the center of the HPT disc samples.

Nanohardness (GPa)	P/M alloys (1250 °C)		HPT	
composition	β	α + β zone	R/2	R
Ti35Nb10Ta	3.7 ± 0.6	4.2 ± 0.6	5.1 ± 0.1	4.8 ± 0.1
Ti35Nb10Ta3Fe	3.3 ± 0.3	5.2 ± 0.5	6.1 ± 0.1	6.1 ± 0.1
**E (GPa)**	**P/M alloys (1250 °C)**		**HPT**	
**composition**	**β**	**α + β zone**	**R/2**	**R**
Ti35Nb10Ta	82 ± 8	91 ± 6	84 ± 2	79 ± 2
Ti35Nb10Ta3Fe	98 ± 3	106 ± 6	84 ± 1	92 ± 1

Table 2 clarifies the greater influence of the various Fe additions on the hardness and elastic modulus in the Ti35Nb10Ta alloy. Moreover, there is a decreasing tendency for the elastic modulus induced by the HPT process due to the nanocrystalline grains obtained by SPD, resulting in a much more uniform and refined nanostructure distribution. The nanohardness (GPa) increases from the stable β phase region to the α + β zones due to the harder α-Ti (hcp) fraction. Additionally, Fe addition seems to stabilize more of the β-Ti phase (minimizing ω phase precipitation), decreasing the hardness from 3.7 to 3.3 GPa. On the other hand, the hardness increases in solid solution and during deformation by the HPT process, changing the hardness values from ~5 GPa to 6.1 GPa for Ti35Nb10Ta3Fe. It is worth noting that the nanohardness and elastic modulus values at the R/2 and R regions of HPT disc sample did not show great variation. It is well known^13,14^ that the torsion strain degree is proportional to radius R, and therefore, the microstructure at the center and periphery of the HPT sample could be non-uniform. Most likely, increasing the rotation number to 5 allowed to avoid this non-uniformity and minimize it.

## Conclusions

- The grain sizes of the P/M samples increased by 25% (45 to 60 µm) with 3 wt% Fe addition when compared to Ti35Nb10Ta, leading to greater β-Ti phase stabilization.
- The application of HPT on the P/M samples generated nanostructured β-Ti alloys without porosity and with a grain size of approximately 50 nm. The occurrence of  nanometric metastable ω phase was confirmed in the β-Ti phase using TEM analysis for the P/M samples (SAD pattern) and HPT samples (virtual dark field - VDF using ASTAR).
- Crystallographic mapping through the ASTAR technique was able to characterize and identify submicron down to nanometric α phase precipitates in the P/M samples as well as characterize nanograins of β-Ti and nanostructured α and ω phase precipitates after HPT, representing a powerful characterization tool for β-Ti and nanostructured alloys.
- The HPT process led to nanostructured β-Ti grains and lowered the elastic modulus (down to 79 and 84 GPa) when compared to the precursor P/M samples (82 and 98 GPa) of the Ti35Nb10Ta and Ti35Nb10Ta3Fe alloys, respectively.
